# Non-conformities in the Pre-analytical Phase at the Parasitology-Mycology Laboratory of the Mohammed VI University Hospital in Oujda

**DOI:** 10.7759/cureus.62632

**Published:** 2024-06-18

**Authors:** Oumaima Nassiri, Fatiha Bousnina, Aziza Hami

**Affiliations:** 1 Central Laboratory, Mohammed VI Hospital University Center, Oujda, MAR

**Keywords:** clinical mycology, clinical parasitology, pre-analytical phase, laboratory, non-conformities

## Abstract

Introduction: The Parasitology-Mycology Laboratory's analytical process involves three stages: pre-analytical, analytical, and post-analytical. Our focus is on the pre-analytical phase (PAP). This study addresses managing PAP non-conformities at Mohammed VI University Hospital in Oujda, aligning with quality standards like ISO 15189 and GBEA and aiming to detect and resolve deviations.

Methods: This 84-month retrospective study analyzed specimens at the Parasitology-Mycology lab in the Mohammed VI University Hospital in Oujda. Examination requests were made through the hospital's IT system (HOSIX), and samples were transported pneumatically. After administrative and technical checks, samples were rejected, processed, or retained for correction based on findings. Reports of non-conformities were sent to prescribers via the IT system. Data were analyzed and flowcharts were created using Microsoft Excel (Redmond, USA).

Results and discussion: During the study period, prescription errors were the most common non-conformities (65.88%; n=56), followed by sample nature errors (29.41%; n=25) and sample packaging errors (4.70%; n=4). Prescription discrepancies, mycological exams for patients on antifungal treatment or carrying Henna, and missing clinical information were the main causes. Outpatient samples accounted for 29.41% of non-conformities, while inpatient samples accounted for 70.59%. The majority of inpatient non-conformities came from the dermatology department (n=42; 49.41%). The pre-analytical phase in Parasitology-Mycology is crucial for ensuring accurate results, involving the coordination of various stages such as staff training, documentation, and non-conformity management. Prescription errors were predominant among non-conformities, followed by sample nature and packaging errors. Outpatient samples had fewer non-conformities compared to inpatient ones, possibly due to supervision by a biologist. Non-conformities lead to therapeutic, prognostic, and economic issues, underscoring the need for their reduction. Corrective actions are crucial, along with establishing policies for error detection and control. Potential causes of non-conformities can be analyzed using methods like the 5M approach. Suggestions for improvement include distributing a validated sampling manual, creating electronic test request forms, staff training, ongoing training programs, and regular meetings for information exchange.

Conclusion: The pre-analytical phase in Parasitology-Mycology is crucial, demanding a quality-focused approach for strict adherence to procedures and traceability. Mastery of this phase ensures result reliability.

## Introduction

In the Parasitology-Mycology laboratory, the analytical process takes place in three distinct stages, each influencing the other: the pre-analytical phase, the analytical phase, and the post-analytical phase. The pre-analytical phase (PAP), which is the subject of our study, is an important stage in the laboratory, with repercussions on the analytical and post-analytical phases. This phase is the responsibility of the medical biologist, who must implement the necessary procedures. The pre-analytical phase is the source of 93% of errors and malfunctions affecting analysis results [1]. These PAP errors can have an impact on the reliability of the results. This underlines the interest of our study on the management of non-conformities in the pre-analytical process within the Parasitology-Mycology laboratory of the Mohammed VI University Hospital in Oujda. This management is a fundamental issue in the implementation of a quality approach that is perfectly in line with the requirements of quality standards, in particular, ISO 15189 and the GBEA. The present study aims to take stock of the existing situation, detect and deal with deviations from quality system requirements, and propose possible solutions.

## Materials and methods

A retrospective study was designed, covering a period of 84 months (seven years) from June 2017 to June 2023. This timeframe was chosen because it corresponds to the establishment of the laboratory seven years ago, and thus, data were collected from its inception. The study encompassed all samples sent to the Parasitology-Mycology laboratory of the Mohammed VI University Hospital Center in Oujda. A total of 7615 requests for parasitological and mycological examinations were gathered. Examination requests were processed using the hospital's computerized system (HOSIX). Samples and prescriptions were sent to the laboratory via the pneumatic system. Upon arrival at the laboratory, secretaries responsible for record-keeping checked for any administrative non-conformities before forwarding them to the technical area. At this stage, technicians and residents ensured there were no non-conformities regarding the sample, its packaging, or the prescription (Table 1). Depending on the identified non-conformities (NC), the sample was either rejected, processed with the identified non-conformity noted, or retained at the laboratory until the non-conformity was rectified. In all cases, a non-conformity report was sent to the prescriber via the laboratory's computer system. All non-conformities stored in our computer system were collected, and no exclusion criteria were applied. Subsequently, the data were entered into Microsoft Excel for analysis and graph creation. Approval was obtained from the department head before utilizing the computer system data. Non-conformities were analyzed using the 5M method [2].

**Table 1:** Criteria for non-conformity of samples

Source of non-conformity	Circumstances
Biological sample	Absent (not sent)
	Empty container
	Insufficient quantity
	Inappropriate for prescribed examination
	Visibly contaminated
	Addressed to the laboratory outside working hours
Packaging	Unsuitable container
	Deteriorated (damaged or leaking)
	Missing, illegible or incomplete label
	Transport time not respected
	Transport temperature not respected
	Damaged equipment
Prescription sheet	Absent
	Patient identity missing. Illegible or incomplete
	Prescriber's identity missing, illegible or incomplete
	Identity of the sampler missing, illegible or incomplete
	Insufficient information
	Sample/prescription sheet mismatch
	Incorrect date

## Results

During the study period, we collected 7615 requests for parasitological and mycological examinations. The overall rate of non-conformities identified during the study period was 1.11%. The majority of non-conformities recorded were linked to prescription errors (65.88%; n=56), followed by errors linked to the nature of the sample (29.41%; n=25), and finally, sample packaging errors (4.70%; n= 4). Among the causes of non-conformity relating to the prescription, we noted mainly the discrepancy between the prescription sheet and the sample (38.82%; n=33); the mycological examination was prescribed for patients undergoing antifungal treatment or carrying Henna (Lawsonia inermis) (24.70%; n= 21), and the clinical information was missing in 2.35% (n= 2) of the cases.

According to the analysis of statistics by the department of origin, the percentage of NC in outpatient samples was 29.41%, compared with 70.59% in inpatient samples.

The majority of NC from inpatient departments came from the dermatology department (n =42; 49.41%) (Table 2). 

**Table 2:** Distribution of non-conformities according to department of origin

Department	Number of non-conformities	Percentage
Dermatology	42	49.41%
External	25	29.41%
Endocrinology	6	7.05%
Gastroenterology	3	3.52%
Neurology	2	2.35 %
Rheumatology	2	2.35%
Neurosurgery	1	1.17%
Internal medicine	1	1.17%
Pediatrics	1	1.17%
Traumatology B	1	1.17%
Reconstructive surgery	1	1.17%

## Discussion

The PAP in Parasitology-Mycology comprises several stages, which must be seamlessly sequenced and coordinated [2]. During this phase, all steps must be of optimum quality, as it is crucial to the quality of the results obtained [3]. The PAP must be conducted according to rigorous, formalized practices. There are three essential aspects to the management of this phase: staff training, documentation, and management of non-conformities [2]. However, the fact that a large part of the PAP takes place outside the laboratory and involves several operators makes it difficult to control [4,5].

The overall rate of non-conformities identified during the study period was 1.11%, contrary to a similar study in Madagascar, which found that only 17.55% of analyses were compliant and of good quality [6]. In our study, the majority of NCs were linked to prescription errors (65.88%; n=56), followed by errors linked to the nature of the sample (27.05%; n=23), and finally, sample packaging errors (7.05%; n=6). The percentage of NC for outpatient samples (29.41%) was lower than for inpatient samples (70.59%). This can be explained by the fact that the pre-analytical phase for outpatients took place inside the laboratory under the supervision of a biologist assigned to the sampling cabinet.

NCs always give rise to therapeutic, prognostic, psychological, and economic problems [7,8]. To combat the latter, it is necessary to reduce NC to its lowest level by improving the PAP. Despite the complexity of the task, the management of this phase remains the sole responsibility of the biologist [9]. ISO 15189 emphasizes the importance of a sampling manual in the documentation system of a medical laboratory. This manual must contain instructions concerning the type and quantity of specimen to be collected, the precise time at which collection is to be carried out as well as any need for special handling between the time of collection and the time of receipt by the laboratory (transport requirements, refrigeration, heating, immediate delivery, etc.); the necessary clinical information; and the safe disposal of materials used for collection. Furthermore, to combat unjustified prescriptions, prescribers should complete an electronic form for requesting Parasitology-Mycology examinations. The latter covers the clinical information needed to guide the choice of test method and the interpretation of results. The biologist must also ensure that staff are familiar with these documents and that they are trained in the use of the equipment if they are to carry out the sampling themselves [10].

Moreover, it is not enough to detect a non-conformity; it must be corrected, the causes investigated, the associated risks assessed, and preventive and improvement actions undertaken, with verification of their effectiveness [11]. To ensure the management of the PAP, it is important, first and foremost, to define a policy for the detection and control of errors [12]. Table 3 shows the various potential causes of NC in the pre-analytical process using the 5M method.

**Table 3:** Analysis of non-conformities using the 5M method

The 5M	Potential causes
Workforce	Multiple contributors: doctor, nurse, courier, technician, biologist
	Workload
	Forget
	Negligence
	Lack of awareness and control
	Lack of training and qualification
	Lack of experience
Methods	Absence of written documents specifying the procedures for patient preparation, sampling transport, storage, centrifugation, volumes to be sampled
Material (sample)	Uncooperative patient
	Patient difficult to prick
	Commensal flora
	Additives
	Sample not representative of infection
Hardware	Out of stock
	Non-sterile material
	Expired tubes
	Incorrect waste management
Environment	Unsuitable sampling room: lighting, air conditioning, heating, etc.
	Hygiene not respected
	Uncontrolled temperatures in storage chambers

Although we were limited by the small number of samples, as this is a new laboratory where the flow is gradually becoming established, we were able to formulate suggestions for improvement in light of our results: Distribute the biological sampling manual validated by the laboratory's head, and regularly update the list of biological analyses available in the hospital laboratory. Also, create electronic analysis request forms to limit unjustified prescriptions. Train and sensitize all staff (prescribers, ward nurses, etc.) to the requirements of the pre-analytical phase, in particular sampling techniques and procedures for routing and transporting biological samples to the laboratory. A system of continuous training should also be set up to qualify all laboratory staff, whatever their grade, in the sorting and control of non-conformities in biological samples. Finally, regular meetings open to all staff and with clinical departments should be organized to enable the exchange of information.

## Conclusions

A detailed analysis of the PAP in Parasitology-Mycology clearly shows that all stages can be critical. Controlling this phase requires a quality approach that brings greater rigor to working methods, a better definition of responsibilities, and harmonization of practices. Eliminating the risk of non-conformity is an essential objective, to which the application of procedures and awareness of the importance of traceability make a major contribution. In this way, mastery of the PAP will guarantee the reliability of the result.
